# Leisure patterns and happiness among Chinese older adults: the moderating effect of age

**DOI:** 10.3389/fpsyg.2025.1540489

**Published:** 2025-05-21

**Authors:** Junyue Yue, Haijun Hao, Yeong Hun Yeo

**Affiliations:** ^1^Department of Social Welfare, Jeonbuk National University, Jeonju, Republic of Korea; ^2^School of Public Administration (School of Philanthropy), Shandong Technology and Business University, Yantai, China

**Keywords:** happiness, leisure patterns, Chinese older adults, age, latent profile analysis

## Abstract

**Introduction:**

As life expectancy rises, leisure activities play a more important role in happiness among older adults. This study aims to identify leisure patterns among Chinese older adults and their relationship with happiness. Further, the changes in the effect of leisure patterns on happiness by age were examined.

**Methods:**

This study used data from the 2018 Chinese General Social Survey (CGSS). We employed a latent profile analysis to clarify leisure patterns of Chinese older adults and hierarchical ordered logistic regression to analyze the relationship between leisure patterns and happiness, as well as the moderating role of age.

**Results:**

First, latent profile analysis identified four leisure patterns: Low Overall Leisure, Exercise-Centered Leisure, Network-Centered Leisure, and High Overall Leisure. Second, the Low Overall Leisure and Network-Centered Leisure groups were associated with lower happiness compared to the High Overall Leisure group. Finally, as age increased, the Low Overall Leisure and the Exercise-Centered leisure groups were progressively associated with higher happiness.

**Conclusion:**

We suggest that when designing leisure activity programs for older adults, full consideration should be given to the leisure needs of different age groups, and programs should be tailored to promote the happiness of the elderly.

## 1 Introduction

With advances in medical technology, global life expectancy continues to rise. By 2050, it is expected to reach an average of 77.2 years (United Nations, 2022). In China, life expectancy has increased from 71.6 years in 2000 to 77.6 years in 2021 (World Health Organization, 2021). This trend suggests that older adults in China will enjoy longer lives. However, as population aging intensifies, the number of people aged 60 and above in China had approached 300 million by the end of 2023 (National Bureau of Statistics of China, 2024). Addressing the challenges of population aging and promoting the happiness of the elderly have become pressing issues in Chinese society today.

Happiness is often defined as an individual's positive evaluation of their overall quality of life and is considered a key indicator of life quality (Veenhoven, 2012; Voukelatou et al., 2021). In later life, leisure activities are viewed as one of the primary sources of happiness for older adults (Kleiber, 2016; Kuykendall et al., 2015) and contribute to active and successful aging (Ryu and Heo, 2018; Marzo et al., 2023). Leisure activities refer to preferred activities individuals engage in during their free time (Kleiber, 2016). Studies have shown that participation in leisure activities helps reduce depressive symptoms (Bone et al., 2022), decrease dementia risk (Su et al., 2022), maintain physical health (Sala et al., 2019), and increase life satisfaction (Cho et al., 2018). A study from Spain also suggests that leisure activities play a key role in determining the level of physical activity in older adults, with games and visiting friends, in particular, being recognized as effective indirect ways of increasing physical activity (Parra-Rizo et al., 2022). Moreover, leisure activities foster social connections among older adults (Hennessy, 2024) and enhance their sense of meaning in life (Iso-Ahola and Baumeister, 2023).

However, although leisure activities play a significant role in enhancing happiness and wellbeing among older adults, understanding the diversity of leisure patterns and their impact on happiness requires more nuanced analytical methods. Traditional studies often focus on specific types of leisure activities (Wei et al., 2015; Ryu and Heo, 2018; Yamashita et al., 2019) or participation frequency (Ku et al., 2016; Paggi et al., 2016), but these approaches may overlook individual differences and lead to the homogenization of leisure experiences. In recent years, Western studies have increasingly adopted an individual-centered perspective rather than a variable-centered approach to examine older adults' participation in leisure activities. For instance, Michèle et al. (2017) used cluster analysis to categorize the leisure patterns of French older adults into seven types; Geithner and Wagner (2022) employed Latent Profile Analysis (LPA) to classify the 14 leisure activities engaged in by German older adults into three patterns. In particular, latent profile analysis is considered an effective method for revealing individual differences in greater detail (Howard and Hoffman, 2018). However, research on the leisure patterns of Chinese older adults remains relatively limited. Zhu et al. (2023) used latent profile analysis to identify three leisure patterns—relaxation, recreation, and intellectual learning—among Chinese older adults. However, their study primarily focused on the oldest age group and examined the relationship between leisure patterns and functional impairment. As a result, it remains unclear how different leisure patterns specifically influence the happiness of older adults. In contrast, the present study employs a broader sample and aims to explore the potential impact of various leisure patterns on the happiness of older adults across all age groups. This aspect has not been sufficiently addressed in the existing literature.

In addition, most studies suggest that participation in leisure activities contributes to improved happiness among older adults (Adams et al., 2011; Ryu and Heo, 2018), but individual characteristics or leisure needs may influence this relationship. For instance, a study from China found that passive leisure activities (e.g., watching television or surfing the Internet) were associated with higher wellbeing than active leisure activities among Chinese residents (Wei et al., 2015). In addition, the relationship between leisure activities and happiness is not entirely linear. Lee et al. (2020) found that the relationship between leisure activities and happiness is complex; excessive participation in leisure activities may reduce leisure satisfaction and happiness. Similarly, Li et al. (2021) identified both linear and curvilinear relationships between the level of leisure participation and its benefits, finding that individuals do not experience optimal leisure benefits when their participation is either too low or too high. In particular, as the aging process progresses, the factors influencing older adults' participation in leisure activities (e.g., physical functioning and residential environment) change (Paillard-Borg et al., 2009; Geithner and Wagner, 2022), leading to changes in the type, amount, and frequency of leisure activities (Strain et al., 2002; Janke et al., 2006; Feng et al., 2020). Older adults may increasingly engage in cognitive and sedentary activities (Finkel et al., 2018), while their participation in physical and social activities has decreased (Fong et al., 2022).

This phenomenon can be explained by the theory of Selection, Optimization, and Compensation (SOC). SOC theory posits that successful personal development involves three components: selection, optimization, and compensation. The realization of these adaptive strategies depends on the specific personal and social environmental changes individuals encounter as they age (Baltes and Baltes, 1990). In response to challenges related to health, economic resources, and changes in the external environment, older adults may choose leisure patterns that better suit their needs. By optimizing the intensity and frequency of activities, they can compensate for the functional losses associated with aging (Freund and Baltes, 1998; Burnett-Wolle and Godbey, 2007). This theory is commonly used to explain the changes in the types and frequencies of leisure activities that older adults engage in over the lifespan (Adams et al., 2011; Nimrod, 2007). However, it remains unclear whether the relationship between leisure patterns and happiness varies with age. In other words, whether the relationship between leisure patterns and happiness is moderated by age still requires further investigation.

In China, with the rapid development of society, older adults are faced with more diverse leisure choices, ranging from traditional activities such as watching television to emerging cultural, recreational, and digital activities. A recent study has shown that Chinese older adults are actively engaging in multiple types of activities, for example, new types of leisure such as socializing online are developing in parallel with traditional leisure such as walking, tai chi, and watching TV (Fang and Ouyang, 2024). The diversity of leisure activities provides a rich context for applying the SOC model. Older adults can positively impact their health and cognitive functioning by choosing activities based on their interests and abilities and optimizing their participation to maximize benefits (Shen et al., 2024). The SOC model explains how Chinese older adults can adjust their participation in leisure activities through SOC strategies to maintain social connectedness and enhance happiness.

In summary, this study uses latent profile analysis to identify the leisure patterns of older adults in China and explore the relationship between different leisure patterns and happiness based on the SOC model, while examining the moderating role of age in this relationship. This study will advance the understanding of the diversity of leisure activities among older adults and provide empirical evidence to inform the development of targeted leisure activities and elderly welfare policies.

## 2 Method

### 2.1 Data source

The data used in this study come from the China General Social Survey (CGSS), conducted by the China Survey and Data Center at Renmin University of China. It is the earliest national academic survey program in China. The survey employs a multi-stage stratified sampling method to analyze the behavioral patterns and social attitudes of Chinese people by collecting information on their behaviors, attitudes, and basic living conditions. The data are representative and accurately reflect the respondents' situation. Since 2003, the survey has publicly released 11 waves of data, with the latest release being for the year 2021, and these data have been widely used in happiness studies (Zhang et al., 2024).

Considering that the dependent variable in this study is happiness, we chose data from the year 2018 instead of the year 2021. First, due to the questionnaire allocation mechanism, the CGSS project team set two questions related to overall happiness in the 2021 survey, and only 67.97% of the older adults answered questions related to both leisure activities and happiness, and another portion of the sample answered only questions related to the International Social Survey Program (ISSP). As a result, the sample size was significantly reduced, and it was impossible to confirm whether the 1991 sample that answered the happiness question was nationally representative. In contrast, all of the samples in the 2018 survey answered questions related to happiness and leisure activities, resulting in more comprehensive data and a larger sample size. In addition, despite easing the epidemic in China in 2021, COVID-19 may still impact the frequency and type of participation in leisure activities among older adults. The impact of this public crisis cannot be ruled out, while the 2018 survey was not disturbed by COVID-19 and has better data representation. For these reasons, we used 2018 data for our analysis. The total sample size for the 2018 survey was 12,787, of which 4,688 were aged 60 and over. After removing missing values, the final sample size for analysis was 3,865.

### 2.2 Measures

#### 2.2.1 Happiness

Happiness measurement primarily uses single-item and multi-item scales (Veenhoven, 2012). The single-item scale measures happiness by directly asking individuals about their level of happiness. While simple, its reliability has been validated (Voukelatou et al., 2021). Most studies on happiness in China also use single-item questions (Zhang et al., 2024). In the 2018 China General Social Survey, respondents were asked, “Overall, how happy do you feel with your life?” using a 5-point Likert scale, where 1 represents “very unhappy” and 5 represents “very happy.” Higher scores indicate greater happiness.

#### 2.2.2 Leisure patterns

In the 2018 CGSS, participants were explicitly asked about their engagement in leisure activities during their free time in the past year. The question provided 12 options: (1) watching television, (2) going to the movies, (3) shopping, (4) participating in physical exercise, (5) reading books/newspapers/magazines, (6) participating in cultural activities, (7) gathering with relatives who do not live together, (8) gathering with friends, (9) listening to music at home, (10) attending live sports events, (11) doing handicrafts (such as embroidery or woodworking), and (12) using the Internet. Each option had five response categories: (1) Daily, (2) Several times a week, (3) Several times a month, (4) Once a year or less, and (5) Never. First, the responses were reverse-coded into a 0–4 Likert scale, with 0 representing “never participating” and 4 representing “daily participation”. Second, considering that low-participation activities contribute less to the representativeness of the overall sample, they may lead to distorted results in the LPA analysis and fail to accurately reflect the characteristics of leisure activities of older adults. To avoid data confounding and maintain the independence of the activity categories, we excluded the following two activities with participation levels higher than 80% instead of categorizing them: attending live sports events and doing handicrafts (over 80% of respondents reported never participating). After excluding these low-participation activities, this study selected ten types of leisure activities for latent profile analysis.

#### 2.2.3 Covariates

Referring to existing research on happiness, this study includes age, gender, residence, education, marital status, family income, and self-rated health as control variables (Wei et al., 2015; Zhang et al., 2024). Specifically, age is treated as a continuous variable, while gender is coded as 0 = female and 1 = male. Education is categorized as follows: 0 = no education, 1 = elementary school, 2 = middle school, and 3 = high school or higher. Residence is coded as 0 = rural and 1 = urban. Marital status is coded as 0 = single and 1 = married. Self-rated health is assessed on a scale of 1 = poor, 2 = fair, and 3 = good. Family income is log-transformed to address right skewness and approximate normality in distribution.

Regarding age, according to China's Elderly Rights Protection Law, individuals aged 60 or above are considered elderly. Additionally, many studies indicate that older adults aged 85 and above engage in fewer types of leisure activities and at a lower frequency due to factors such as physical decline, which may skew the leisure activity patterns of the entire sample (Agahi et al., 2006; Dodge et al., 2008). Therefore, this study excluded participants aged 85 and older, focusing only on those aged 60–85.

### 2.3 Data analysis

First, descriptive statistical analysis was performed on the total samples, with continuous variables summarized by means (M) and standard deviations (SD), and categorical variables represented by frequencies (N) and percentages (%). Second, latent profile analysis was conducted on the 10 types of leisure activities. Third, analysis of variance (ANOVA) was used for continuous variables, while the Pearson χ^2^ test was used for categorical variables to examine significant differences between different leisure patterns. Finally, ordered logistic regression analysis was used to investigate the relationship between leisure patterns and happiness and to test the moderating effect of age on this relationship. This study conducted latent profile analysis using Mplus 8.3, while all other analyses were performed using Stata 18.0. A *p-*value < 0.05 was considered indicative of statistical significance.

In addition, for the evaluation of model fit in LPA, a decrease in the Akaike Information Criterion (AIC), Bayesian Information Criterion (BIC), and sample-size adjusted BIC (aBIC) values indicates improved model fit as the number of categories increases (Collins et al., 1993; Nylund et al., 2007). The Lo-Mendell-Rubin likelihood ratio test (LMR) and the Bootstrapped Likelihood Ratio Test (BLRT) were used to assess model fit further. Models with *p-*values < 0.05 were considered to fit better with the k-category model than the k-1 category model (Nylund et al., 2007). The entropy index reflects individual classification accuracy; values closer to 1.0 indicate higher classification accuracy, which is generally considered satisfactory (Lubke and Muthén, 2007). We adopted 0.80 as the threshold for entropy, which is widely considered acceptable in gerontological literature (Liu et al., 2023; Zhu et al., 2023). Class probabilities represent the proportion of each classification within the total sample, with values >5% considered appropriate to ensure the stability and robustness of the model.

## 3 Results

### 3.1 Sample characteristics

Table 1 shows the descriptive statistics of the variables in the 2018 CGSS. The majority of respondents were young-old adults (*M* = 68.83, SD = 6.57), female (*N* = 1,987, 51.41%), urban residents (*N* = 2,584, 66.86%), and had completed elementary school (*N* = 1,142, 29.55%). Regarding marital status, most were married (*N* = 2,906, 75.19%), and most respondents rated their health as good (*N* = 1,563, 40.44%). The mean family income was CNY 54,266.62 (SD = 77,576.68). Additionally, the mean happiness score of the respondents was 3.95 (SD = 0.84).

**Table 1:** Descriptive statistics of variables in CGSS 2018 (*N* = 3,865).

**Variables**	**Classification**	***N* (Mean)**	**SD (%)**
Age	60–85	68.83	6.57
Gender	Male	1,878	48.59
	Female	1,987	51.41
Residence	Urban	2,584	66.86
	Rural	1,281	33.14
Education	None	947	24.50
	Elementary school	1,142	29.55
	Middle school	921	23.83
	High school and above	855	22.12
Marital status	Married	2,906	75.19
	Single	959	24.81
Self-rated health	Bad	1,171	30.30
	Fair	1,131	29.26
	Good	1,563	40.44
Family income	0–999,600	54,266.62	77,576.68
Happiness	1–5	3.95	0.84

### 3.2 Latent profile analysis of leisure activities

#### 3.2.1 Indicators for each latent profile of leisure activities

Table 2 presents the results for the five fitted models. The AIC, BIC, and aBIC values decreased progressively across the models. The *p-*value for the BLRT was consistently below 0.05 for all fitted models, while the *p-*value for the LMR in the 5-profile model exceeded 0.05. All fitted models had entropy values greater than 0.9, with the 3-profile model exhibiting the highest entropy. During the model selection process, the 5-profile model was initially excluded because its LMR *p-*value was >0.05. Despite the 3-profile model having the highest entropy, the *p-*values for the LMR and BMIR in the 4-profile model were both below 0.05, indicating that the 4-profile model provided a better fit than the 3-profile model.

**Table 2:** Indicators for each latent profile of leisure activities in older adults.

**Profile**	**AIC**	**BIC**	**aBIC**	**Entropy**	**LMR (P)**	**BLRT (P)**	**Class probabilities**
1c	116,697.22	116,822.42	116,758.87	–	–	–	–
2c	106,876.91	107,070.96	106,972.46	0.994	0.000	0.000	0.744/0.256
3c	104,479.29	104,742.20	104,608.74	0.997	0.000	0.000	0.706/0.059/0.235
4c	101,944.92	102,276.68	102,108.27	0.982	0.000	0.000	0.09/0.486/0.256/0.168
5c	99,591.32	99,991.94	99,788.58	0.996	0.189	0.000	0.082/0.706/0.035/0.159/0.018

AIC, Akaike Information Criterion; BIC, Bayesian Information Criterion; aBIC, Adjusted Bayesian Information Criteria; LMR, Lo-Mendell-Rubin; BLRT, Bootstrapped likelihood ratio test.

Moreover, the 3-profile model showed substantial discrepancies in the probabilities assigned to each class, with the smallest class accounting for only 5.9%. In contrast, the class probabilities in the 4-profile model appeared more balanced and reasonable. Considering these factors, the 4-profile model was ultimately the most appropriate.

#### 3.2.2 Naming of leisure patterns

**Figure 1:**
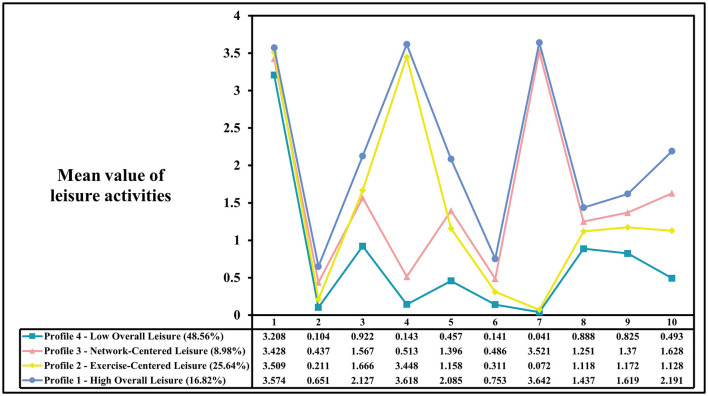
Latent profile model of leisure activities in Chinese older adults. (1) watching television, (2) going out to the movies, (3) shopping, (4) participating in physical exercise, (5) reading books/newspapers/magazines, (6) participating in cultural activities, (7) using the Internet, (8) gathering with relatives who don't live together, (9) gathering with friends, (10) listening to music at home.

Figure 1 shows the results of the latent profile analysis of ten leisure activities among older adults in China. Overall, the four patterns exhibit three common characteristics: high participation frequency in TV watching, low participation frequency in cultural activities (such as going to the movies or attending cultural events), and moderate participation frequency in social gatherings (with relatives or friends).

Specifically, the sample in “Profile 1” scored higher than the other profiles across all categories, reflecting the fact that older adults with this profile engage more in leisure activities overall. Therefore, this profile was named “High Overall Leisure” (16.82%). The sample in “Profile 2” scored higher in physical activity, just below “Profile 1,” but lower in Internet use and social activities. This suggests that the leisure pattern of the elderly in this group is exercise-centered, and thus it was named “Exercise-Centered Leisure” (25.64%). The sample in “Profile 3” scored higher in Internet use and socializing with relatives and friends, second only to “Profile 4,” indicating a leisure pattern centered around both online and offline social networks. Hence, this profile was named “Network-Centered Leisure” (8.98%). Finally, the sample in “Profile 4” scored lower than the other patterns in all items, with only TV watching scoring higher. This indicates an overall lower level of leisure engagement, and it was named “Low Overall Leisure” (48.56%).

#### 3.2.3 Characteristic differences in leisure patterns

Table 3 presents a comparison of demographic characteristics and happiness differences across the various leisure patterns. The results showed significant differences in all variables, except gender, across the four leisure patterns, with *p-*values < 0.001. Specifically, older adults living in urban areas and those who are married had higher participation rates in leisure activities compared to their rural counterparts and singles.

**Table 3:** Characteristic differences in leisure patterns.

**Variables**	**Low overall leisure (*N =* 1,877)**	**Network-center leisure (*N =* 347)**	**Exercise-center leisure (*N =* 991)**	**High overall leisure (*N =* 650)**	***F*/χ^2^**	***P***
Age	69.72 (6.75)	66.37 (5.69)	69.63 (6.66)	66.33 (5.28)	67.33	0.000
Gender					4.80	0.187
Male	884 (47.10)	164 (47.26)	496 (50.05)	334 (51.38)		
Female	993 (52.90)	183 (52.74)	495 (49.95)	316 (48.62)		
Residence					485.14	0.000
Urban	959 (51.09)	285 (82.13)	728 (73.46)	612 (94.15)		
Rural	918 (48.91)	62 (17.87)	263 (26.54)	38 (5.85)		
Education					1,500	0.000
None	698 (37.19)	24 (6.92)	205 (20.69)	20 (3.08)		
Elementary school	686 (36.55)	63 (18.16)	339 (34.21)	54 (8.31)		
Middle school	320 (17.05)	104 (29.97)	272 (27.45)	225 (34.62)		
High school and above	173 (9.22)	156 (44.96)	175 (17.66)	351 (54.00)		
Marital status					58.49	0.000
Married	1,336 (71.18)	299 (86.17)	734 (74.07)	537 (82.62)		
Single	541 (28.82)	48 (13.83)	257 (25.93)	113 (17.38)		
Self-rated health					230.59	0.000
Bad	769 (40.97)	69 (19.88)	248 (25.03)	85 (13.08)		
Fair	499 (26.58)	111 (31.99)	291 (29.36)	230 (35.38)		
Good	609 (32.45)	167 (48.13)	452 (45.61)	335 (51.54)		
Family income (CNY)	34,429.67 (53,381.03)	83,710.09 (101,905.5)	56,689.33 (87,746.87)	92,137.63 (85,238.94)	119.62	0.000
Happiness	3.86 (0.87)	3.90 (0.87)	4.07 (0.80)	4.07 (0.74)	19.38	0.000

The values represent the sample sizes (%) or sample means (SD). SD, standard deviation.

Among four profiles, samples in the Low Overall Leisure group had the highest average age (*M* = 69.72 years), were predominantly female, uneducated, and in poor health. In contrast, samples in the High Overall Leisure group had the lowest average age (*M* = 66.33 years), were mostly male, highly educated, and in good health. Compared to the High Overall Leisure group, samples in the Exercise-Centered Leisure group also reported good health but had a higher average age (*M* = 69.63 years), were mostly male, and had only elementary education. Samples in the Network-Centered Leisure group had an average age of 66.37 years, were primarily female, had higher education, and reported good health. In terms of income, older adults in the High Overall Leisure group reported the highest family income, followed by those in the Network-Centered Leisure group. In contrast, participants in the Exercise-Centered Leisure and Low Overall Leisure groups reported the lowest income levels.

Regarding happiness scores, the High Overall Leisure and Exercise-Centered Leisure groups exhibited the highest happiness levels, followed by the Network-Centered Leisure and Low Overall Leisure groups.

### 3.3 Ordered logistic regression of leisure patterns, age, and happiness

Before conducting ordered logistic regression analyses, we conducted multinomial regression analyses to explore the factors influencing leisure patterns among Chinese older adults. Specific results are presented in Supplementary Table S1. The analyses showed that older adults who lived in rural areas, had no formal education, had no spouse, were in poorer health, and had lower household economic status were more likely to be categorized as having the Low Overall Leisure pattern than any other leisure pattern. Table 4 presents the results of the ordered logistic regression analyzing the relationship between leisure patterns and happiness. In Model 1, all control variables, except for residence and education, significantly predicted happiness among older adults. Model 2 built upon Model 1 by adding leisure patterns, showing that compared to the High Overall Leisure, the Network-Centered Leisure (OR = 0.73, 95% CI = 0.56–0.94) and the Low Overall Leisure (OR = 0.79, 95% CI = 0.64–0.98) were associated with lower happiness. Whereas Exercise-Centered Leisure was associated with higher happiness, but did not reach statistical significance. Model 3 examined the moderating effect of age on the relationship between leisure patterns and happiness. The results indicated that age positively moderates the relationship between leisure patterns and happiness, using High Overall Leisure as the reference group. Specifically, as older adults age, Low Overall Leisure (OR = 1.039, 95% CI = 1.01–1.07) and Exercise-Centered Leisure (OR = 1.037, CI = 1.00–1.07) were associated with higher levels of happiness compared to High Overall Leisure.

**Table 4:** Ordered logistic regression of leisure patterns, age, and happiness.

**Variables**	**Model 1**		**Model 2**		**Model 3**	
	**OR**	**95% CI**	**OR**	**95% CI**	**OR**	**95% CI**
Age	1.04^***^	(1.03, 1.05)	1.04^***^	(1.03, 1.05)	1.01	(0.98, 1.04)
**Gender (ref. female)**						
Male	0.81^**^	(0.71, 0.93)	0.81^**^	(0.71, 0.92)	0.81^**^	(0.71, 0.93)
**Education (ref. none)**						
Elementary school	1.17	(0.98, 1.39)	1.14	(0.95, 1.35)	1.14	(0.95, 1.35)
Middle school	1.20	(0.99, 1.46)	1.14	(0.94, 1.40)	1.15	(0.94, 1.40)
High school and above	1.14	(0.93, 1.40)	1.10	(0.88, 1.37)	1.10	(0.88, 1.37)
**Marital status (ref. single)**						
Married	1.35^***^	(1.16, 1.57)	1.361^***^	(1.17, 1.59)	1.38^***^	(1.18, 1.61)
**Residence (ref. rural)**						
Urban	0.97	(0.83, 1.12)	0.92	(0.79, 1.08)	0.92	(0.79, 1.07)
**Self-rated health (ref. bad)**						
Fair	1.53^***^	(1.29, 1.80)	1.49^***^	(1.26, 1.77)	1.50^***^	(1.27, 1.77)
Good	2.57^***^	(2.19, 3.01)	2.48^***^	(2.12, 2.91)	2.49^***^	(2.12, 2.92)
Family income	1.09^***^	(1.06, 1.12)	1.09^***^	(1.06, 1.12)	1.09^***^	(1.06, 1.12)
**HOL (ref)**						
LOL			0.79^*^	(0.64, 0.98)	0.06^**^	(0.01, 0.51)
ECL			1.13	(0.92, 1.40)	0.10	(0.01, 1.02)
NCL			0.73^*^	(0.56, 0.94)	0.07^**^	(0.00, 1.54)
Age^*^LOL					1.039^*^	(1.01, 1.07)
Age^*^ECL					1.037^*^	(1.00, 1.07)
Age^*^NCL					1.036	(0.99, 1.08)
Log-likelihood	−4,214.34		−4,201.65		−4,198.74	
LR chi-square	273.65^***^		299.03^***^		304.86^***^	

^*^p < 0.05, ^**^p < 0.01, ^***^p < 0.001.

HOL, High Overall Leisure; LOL, Low Overall Leisure; ECL, Exercise-Centered Leisure; NCL, Network-Centered Leisure; OR, odds ratio; 95% CI, 95% confidential intervals.

**Figure 2:**
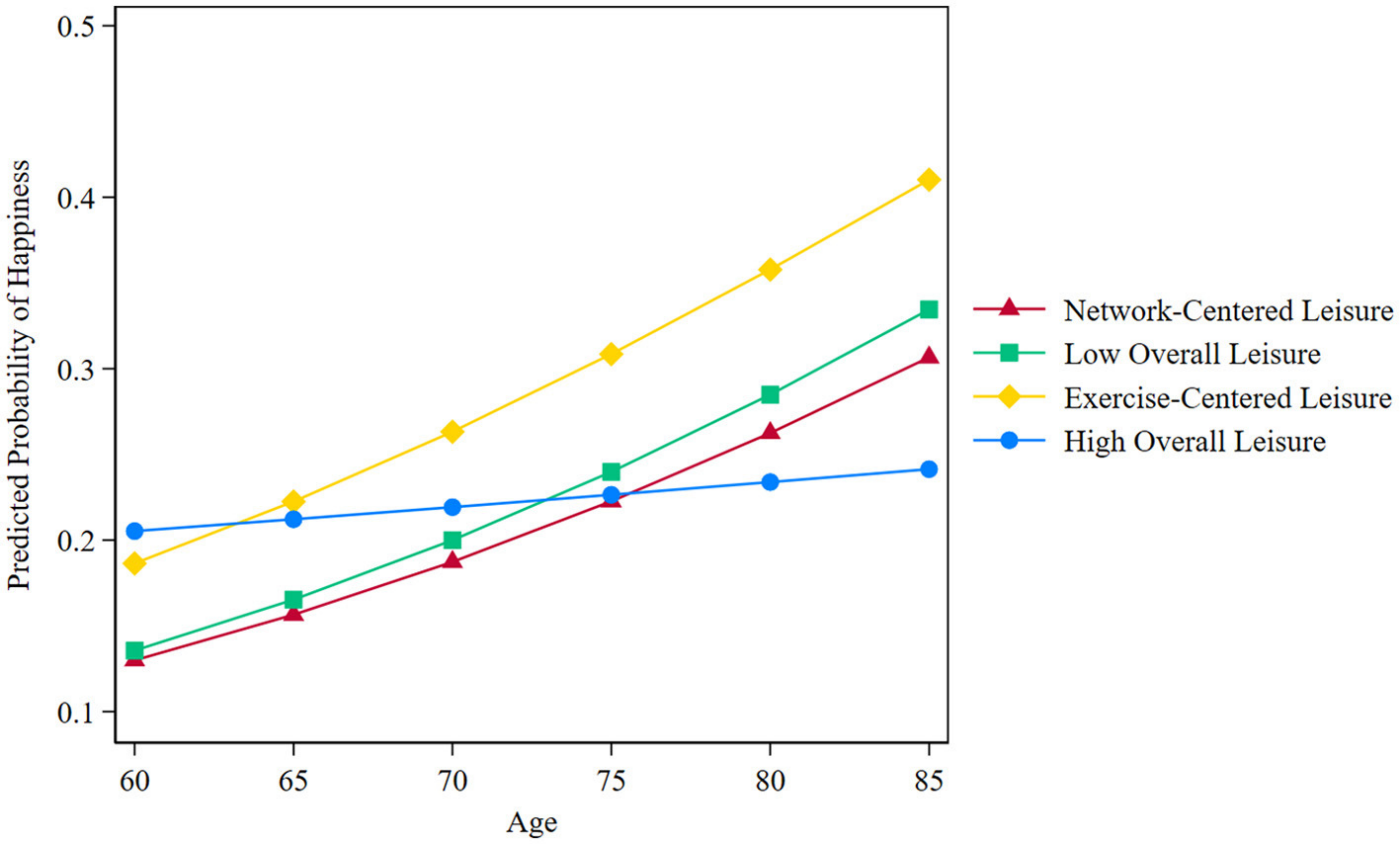
The moderating role of age between leisure patterns and happiness.

Figure 2 shows the moderating effect of age on the relationship between leisure patterns and happiness. Overall, there is a positive relationship between leisure patterns and happiness. However, as age increases, different leisure patterns predict the happiness levels of Chinese older adults differently. The predicted happiness values for Low Overall Leisure, Network-Centered Leisure, and Exercise-Centered Leisure increase significantly with age, while the predicted happiness value for High Overall Leisure increases only slightly. According to the predicted values (see Supplementary Table S2), between ages 60–65, High Overall Leisure has the highest predicted happiness among the four leisure patterns. However, after the age of 65, Exercise-Centered Leisure has the highest predicted happiness value, followed by Low Overall Leisure and Network-Centered Leisure. In addition, we found that the predictive value of the High Overall Leisure for happiness remained high when the elderly were aged up to 75 years, but after reaching 75 years, the predictive value of the High Overall Leisure for happiness became the lowest scoring of the four patterns.

## 4 Discussion

To understand the leisure patterns of Chinese older adults and explore the relationship between these patterns and happiness, we first identified four distinct leisure patterns using latent profile analysis: Low Overall Leisure (48.6%), Exercise-Centered Leisure (25.6%), Network-Centered Leisure (9%), and High Overall Leisure (16.8%). Next, we examined the relationship between these leisure patterns and happiness among older adults through ordered logistic regression analysis, further investigating the moderating effect of age on this relationship.

First, the four leisure patterns showed significant differences in age, residence, education level, marital status, self-rated health, and household income, with no significant differences found in gender. Specifically, the Low Overall Leisure group had the oldest average age (69.72 years), predominantly female, low education, poor self-rated health, and the lowest household income. The High Overall Leisure group was the youngest (66.3 years), primarily male, highly educated, in good health, and had the highest household income. The Exercise-Centered Leisure group had an average age of 69.63 years, male, with middle school education, good self-rated health, and household income higher than the Low Overall Leisure group but lower than the High Overall Leisure group. The Network-Centered Leisure group had an average age of 66.37 years, was mainly female, had high school education, good self-rated health, and household income second only to the High Overall Leisure group. Across all groups, most older adults lived in cities and were married. These demographic differences align with prior studies, further confirming the impact of social and personal factors on leisure participation (Paillard-Borg et al., 2009; Finkel et al., 2018; Geithner and Wagner, 2022).

We found that the Low Overall Leisure pattern is dominant among Chinese older adults, indicating that leisure participation remains low and requires urgent improvement, particularly among those with poorer health and economic status. Many older adults reduce their engagement in high-intensity activities due to health issues, opting instead for low-intensity activities such as watching TV or socializing with friends (Feng et al., 2020; Finkel et al., 2018). Furthermore, although the average age of the Exercise-Centered Leisure group was similar to that of the Low Overall Leisure group, older adults in the Exercise-Centered Leisure group generally reported better health, comparable to those in the High Overall Leisure group. These findings suggest that regular exercise may help maintain good health, even in advanced age. As Paggi et al. (2016) observed, reductions in leisure activity are more closely related to physical health limitations than aging.

Low leisure engagement among the Low Overall Leisure group may be attributed to several interrelated factors. First, limited physical functioning and chronic health conditions often act as primary barriers, reducing older adults' ability or willingness to participate in diverse leisure activities (Paggi et al., 2016; Finkel et al., 2018). Second, socio-demographic factors such as lower education levels, rural residence, and lower income are significantly associated with reduced leisure participation. As shown in our study, older adults in the Low Overall Leisure group were more likely to live in rural areas, have no formal education, and report poorer health, which aligns with findings from previous research on structural inequalities in leisure engagement (Chen and Tsai, 2020; Paillard-Borg et al., 2009).

Moreover, psychological factors such as lack of motivation or social isolation may further limit engagement in leisure activities (Hennessy, 2024). Some older adults may also lack awareness of or access to leisure opportunities tailored to their preferences or capabilities. This highlights the need for targeted interventions. Intervention programs should prioritize identifying and engaging older adults with these risk factors. For instance, community-based programs could provide transportation, health support, or subsidized access to cultural and recreational facilities, particularly in rural or underserved areas (Geithner and Wagner, 2022). Additionally, policies aimed at improving health literacy and fostering inclusive, low-barrier activities such as intergenerational events or home-based physical activities may empower older adults with limited mobility to remain engaged (Fancourt et al., 2021).

Second, regarding happiness scores, the ranking was as follows: High Overall Leisure, Exercise-Centered Leisure, Network-Centered Leisure, and Low Overall Leisure. This finding aligns with existing literature suggesting that active participation in leisure activities provides more significant benefits to older adults, while limited engagement in leisure activities may be associated with poorer outcomes (Adams et al., 2011; Wei et al., 2015; Cho et al., 2018; Yamashita et al., 2019). It is worth noting, however, that older adults in the Network-Centered Leisure group reported lower happiness, which is not entirely consistent with the prevailing view in the literature that social activities generally contribute to the happiness of older adults. Most studies suggest that older adults who engage more in social networks, whether online or offline, should experience higher happiness than those with limited leisure participation. However, our study found that the Network-Centered Leisure group had a higher happiness score than the Low Overall Leisure group but lower than the High Overall Leisure and Exercise-Centered Leisure groups. A distinctive feature of the Network-Centered Leisure group is Internet use.

Studies on Internet use among older adults have shown that the most common Internet activities used by Chinese older adults are communicating with others or having fun through social media (e.g., WeChat and TikTok) (Sun et al., 2020; Tang et al., 2022). Although some studies have shown that Internet use can provide social support to older adults, not all forms of online interaction contribute to happiness (Szabo et al., 2018). For instance, some older adults may turn to the Internet for social support due to a lack of real-life social connections (Jia et al., 2022), however, this “compensation” behavior does not significantly enhance their happiness and may even exacerbate loneliness (Tang et al., 2022). Our study suggests that although older adults engage frequently in online leisure activities, these activities may lack emotional depth and primarily serve as a substitute for face-to-face interactions. Consequently, the relatively weak happiness effect of Network-Centered Leisure indicates that online social activities do not always fulfill the emotional needs of older adults.

Finally, the study also found that age significantly moderates the relationship between leisure patterns and happiness. Specifically, for younger older adults in their 60s, the association between High Overall Leisure and happiness was more substantial, with extensive leisure participation being particularly beneficial. However, this effect diminished as age increased. For those over 70, the impact of the High Overall Leisure pattern on happiness significantly weakened, even approaching the lowest happiness probability among all patterns. In contrast, the relationships between Low Overall Leisure, Network-Centered Leisure, and Exercise-Centered Leisure patterns and happiness became more potent with age. This phenomenon can be explained through the theory of Selection, Optimization, and Compensation (SOC). According to SOC theory, in the early stages of aging, older adults can optimize their happiness by actively engaging in various leisure activities. However, as they age, older adults face increasing physical and psychological challenges, leading them to adopt selective and compensatory strategies. These strategies involve choosing activities suitable for their health status, helping them mitigate potential health risks (Gomillion et al., 2016).

Additionally, while some older adults in the Exercise-Centered Leisure group were older, they still reported the highest levels of happiness, likely due to their consistent engagement in physical activity over time. In contrast, the younger individuals in the High Overall Leisure group may have optimized their social and psychological resources by diversifying their activities to cope with the multiple demands of life. However, as they age, the impact of this pattern on happiness diminishes, and even in the oldest group, the effect of High Overall Leisure on happiness weakens, showing the lowest predictive probability. This finding underscores the crucial role of physical activity in promoting the happiness of older adults in later life.

Overall, these findings reveal the different leisure patterns of Chinese older adults and their complex relationship with happiness. They help us better understand how older adults maintain happiness by adapting leisure activities at different life stages and provide strong theoretical support for social participation and health promotion strategies for older adults. In this regard, we suggest that leisure activity not only focuses on the type of activity or the frequency of activity participation, but also that different interventions should be adopted for different leisure patterns and age subgroups.

Specifically, the Low Overall Leisure pattern accounts for nearly half of the older adult population. They are typically associated with health limitations, low income, low education, and rural residence. In order to improve the happiness of this group, intervention strategies should focus on improving accessibility to leisure activities and lowering participation thresholds. For example, enhancing leisure facilities in rural or low-income neighborhoods to provide mobile libraries, cultural events, and social gatherings can help overcome geographic and economic barriers. Free or subsidized activity fees, transportation services, etc., can also be provided, especially adaptive exercise classes for older people with poorer health. The Network-Centered Leisure group may benefit from interventions that enhance the quality of their social interactions. For example, communities can organize social networking events, such as “social media clubs,” to help older adults meet face-to-face, increase digital literacy, and reduce loneliness. For Exercise-Centered and High Overall Leisure groups, intervention strategies should maintain and promote their leisure participation by providing more age-friendly sports programs and cultural activities to help maintain social connections and mental health.

This study has several limitations. First, due to the cross-sectional nature of the data, we were unable to establish causal inferences. Future studies should consider using longitudinal data, such as latent transfer analysis or survival analysis, to explore the dynamic relationship between leisure patterns and happiness more effectively. Second, the range of leisure activities included in this study was limited and did not capture all possible leisure activities; for instance, emerging activities such as fishing and travel were excluded from the analysis. Future research should develop more comprehensive measurement tools to better identify and track the full spectrum of leisure activities among older adults. Third, while we excluded adults aged 85 and older, this decision may limit the generalizability of our findings, particularly in understanding the relationship between leisure activities and happiness in this age group. Future research could address this limitation by adopting alternative methods that include individuals aged 85 and older. Finally, it is important to note that the current analysis did not fully account for all potential confounding variables, especially regarding self-report biases. Self-reported measures of happiness and leisure activities may be influenced by momentary mood or social desirability, which could introduce bias. Future studies should consider combining objective data or informant reports to improve the reliability of conclusions and further enhance the robustness and reliability of the findings.

## 5 Conclusion

This study utilized latent profile analysis to identify leisure patterns among Chinese older adults and explored the relationship between these patterns and happiness while examining the moderating effect of age. The findings offer new insights into understanding the leisure activities of older adults in China and provide valuable guidance for policymakers in crafting leisure and welfare policies for this demographic. In formulating policies on leisure activities for older adults, policymakers should consider the demographic characteristics and social backgrounds of different groups to enhance the overall happiness of older adults. We advocate for encouraging active participation in leisure activities, particularly physical activities centered around exercise. However, age must be carefully considered. As older adults age, they should gradually transition away from High Overall Leisure activities and select leisure activities that are more appropriate for their physical condition. Therefore, in designing relevant policies, it is important to consider the specific needs of older adults in different age groups and to avoid recommending activities that may not be suitable for their physical capabilities.

## Data Availability

Publicly available datasets were analyzed in this study. This data can be found here: http://cgss.ruc.edu.cn.
