# Supplementary material for: Leisure patterns and happiness among Chinese older adults: the moderating effect of age
Source: Front Psychol. 2025 May 21;16:1540489. doi: 10.3389/fpsyg.2025.1540489 (PMC12133746; doi:10.3389/fpsyg.2025.1540489)
Supplement: Supplementary file 1 [file Table_1.docx]

Supplementary Material

# Supplementary Table S1. Analysis of Factors Influencing the Leisure Patterns among Chinese Older Adults.

| **Variables** | **Model 1** | | **Model2** | | **Model3** | |
| --- | --- | --- | --- | --- | --- | --- |
|  | **NCL (ref. LOL)** | | **ECL (ref. LOL)** | | **HOL (ref. LOL)** | |
|  | **OR** | **95% CI** | **OR** | **95% CI** | **OR** | **95% CI** |
| **Age** | 0.92*** | (0.90, 0.94) | 1.00 | (0.99, 1.01) | 0.91*** | (0.89, 0.93) |
| **Gender (Ref. Female)** | 0.59*** | (0.45, 0.76) | 0.91 | (0.77, 1.08) | 0.73** | (0.58, 0.91) |
| **Residence (Ref. Rural)** | 2.02*** | (1.45, 2.83) | 1.91*** | (1.59, 2.30) | 4.43*** | (3.02, 6.51) |
| **Education (Ref. None)** | | | | | | |
| **Elementary school** | 2.80*** | (1.71, 4.58) | 1.59*** | (1.28, 1.96) | 2.57** | (1.50, 4.41) |
| **Middle school** | 7.68*** | (4.72, 12.50) | 2.30*** | (1.81, 2.93) | 13.99*** | (8.49, 23.04) |
| **High school and above** | 18.90*** | (11.54, 30.94) | 2.24*** | (1.76, 3.11) | 30.52*** | (18.33, 50.82) |
| **Marital status (Ref. Single)** | | | | | | |
| **Married** | 1.61** | (1.13, 2.30) | 1.02 | (0.84, 1.23) | 1.00 | (0.75, 1.33) |
| **Self-rated health (Ref. Bad)** | | | | | | |
| **Fair** | 1.64** | (1.16, 2.31) | 1.51*** | (1.22, 1.86) | 2.32*** | (1.69, 3.18) |
| **Good** | 2.16*** | (1.57, 2.99) | 1.99*** | (1.64, 2.42) | 3.05*** | (2.26, 4.12) |
| **Family income (CNY)** | 1.10** | (1.03, 1.18) | 1.06** | (1.02, 1.10) | 1.40*** | (1.27, 1.55) |

^**^ *p* < .01, ^***^ *p* < .001; HOL = High Overall Leisure; LOL = Low Overall Leisure; ECL = Exercise-Centered Leisure; NCL = Network-Centered Leisure; OR, odds ratio; 95% CI, 95% confidential intervals.

# Supplementary Table S1. Predictive probability of leisure patterns on happiness of older adults of different ages.

| **Age** | **Network-Centered Leisure** | **Low Overall leisure** | **Exercise-Centered Leisure** | **High Overall Leisure** |
| --- | --- | --- | --- | --- |
| **60** | 0.130 | 0.136 | 0.186 | **0.205** |
| **65** | 0.156 | 0.165 | **0.222** | **0.212** |
| **70** | 0.187 | 0.200 | **0.263** | **0.219** |
| **75** | 0.223 | **0.240** | **0.309** | 0.226 |
| **80** | 0.262 | **0.285** | **0.358** | 0.234 |
| **85** | 0.307 | **0.334** | **0.410** | 0.241 |
